# An Ultrasound Simulation Model for the Pulsatile Blood Flow Modulated by the Motion of Stenosed Vessel Wall

**DOI:** 10.1155/2016/8502873

**Published:** 2016-07-10

**Authors:** Qinghui Zhang, Yufeng Zhang, Yi Zhou, Kun Zhang, Kexin Zhang, Lian Gao

**Affiliations:** ^1^Department of Electronic Engineering, Information School, Yunnan University, Kunming, Yunnan 650091, China; ^2^School of Computer and Information, Southwest Forestry University, Kunming, Yunnan 650224, China; ^3^School of Information, Yunnan Normal University, Kunming, Yunnan 650500, China; ^4^Cardiovascular Department, The Second Affiliated Hospital of Kunming Medical College, Kunming, Yunnan 650031, China

## Abstract

This paper presents an ultrasound simulation model for pulsatile blood flow, modulated by the motion of a stenosed vessel wall. It aims at generating more realistic ultrasonic signals to provide an environment for evaluating ultrasound signal processing and imaging and a framework for investigating the behaviors of blood flow field modulated by wall motion. This model takes into account fluid-structure interaction, blood pulsatility, stenosis of the vessel, and arterial wall movement caused by surrounding tissue's motion. The axial and radial velocity distributions of blood and the displacement of vessel wall are calculated by solving coupled Navier-Stokes and wall equations. With these obtained values, we made several different phantoms by treating blood and the vessel wall as a group of point scatterers. Then, ultrasound echoed signals from oscillating wall and blood in the axisymmetric stenotic-carotid arteries were computed by ultrasound simulation software, Field II. The results show better consistency with corresponding theoretical values and clinical data and reflect the influence of wall movement on the flow field. It can serve as an effective tool not only for investigating the behavior of blood flow field modulated by wall motion but also for quantitative or qualitative evaluation of new ultrasound imaging technology and estimation method of blood velocity.

## 1. Introduction

Atherosclerosis is one of the most common types of cardiovascular disease and is usually related to the existence of stenosis in an artery. The stenosis is caused by a plaque formed by the accumulation of lipid substances, cholesterol, cellular waste products, calcium, and fibrin in the inner lining of an artery. The plaque may partially or totally make arteries clog up, which finally may result in a heart attack and stroke [1–3]. In addition, it has been well accepted that once a mild stenosis is developed, the resulting flow disorders further influence the development of the disease and arterial deformability and change the regional blood rheology [4]. Thus, efficient imaging of the blood flowing through a stenosed vessel is necessary for the fundamental understanding, early diagnosis, and treatment of many cardiovascular diseases.

As a consequence of technological advances in ultrasound imaging, the ultrasonic echography has become one of the most important noninvasive diagnostic methods for detection and monitoring of cardiovascular disease. Compared with other available imaging methods, it is quick, safe, and relatively inexpensive when visualizing arterial wall and flow in vivo [5]. However, these ultrasound images, such as B-mode, M-mode Doppler, and color flow imaging (CFI) ultrasound images, cannot always accurately describe the real flow behaviors. The weak signals backscattered from the flowing blood, especially the signals echoed from the area near the vessel wall, will be contaminated by the strong backscattered clutter signals originating from stationary tissues, slowly moving vessels, and noise. However, it is believed that the echoed ultrasonic signals from slowly moving blood flow near the wall may provide useful information to improve the diagnostic efficiency.

Thus, many researchers have made remarkable efforts to develop new velocity estimation method, clutter filter, signal processing, and visualization techniques to obtain more accurate signals of blood flow. Therefore, the evaluation of these new techniques is important. It has been generally accepted that the validation using in vivo data is not desirable, because the signal components from the surrounding tissue and blood are not easily distinguishable, and the exact behaviors of the vessel wall are unknown [6]. Conversely, the computer simulation is a useful validation method because all parameters can be well defined.

Hence, some researchers constructed simulation models to imitate the behaviors of pulsatile blood flow based on the assumption that the wall of the artery is rigid [7–10]. Recently, taking into account the interaction between blood flow and the vessel wall, several investigators set up more realistic and complex models with the help of computational fluid dynamics (CFD) software [11–17]. They studied the effects of mechanical parameters of the elastic vessel such as stenosis severity, stenosis geometry, and length on the flow field. In these studies, models were built up considering wall moving only along with blood pulsation. However, it was reported that the motion of the vessel wall is influenced by not only blood pulsation, but also other physiological actions such as heartbeat, breathing, and body posture [6, 18]. These actions will change the movement of the vessel wall, which will interact with blood flow. The interaction is not yet clearly understood and will deteriorate the blood velocity estimation due to the strong echoes from the vessel wall.

More recently, a coupled method integrating the computational fluid dynamics (CFD) and ultrasound simulation was introduced in some studies [19–23]. Investigators modeled blood and vessel as a collection of point scatterers whose positions are updated using the interpolated velocity generated by CFD software. This coupled method provides a better understanding of the relationship between ultrasound images and the actual flow dynamics, as well as a better investigation of blood flow behaviors. However, for the method based on CFD software, setting and changing the boundary and initial conditions are inconvenient, and computing is time-consuming, which restricts its scope of application.

The purpose of this study is to present a simulation model which can generate more realistic ultrasound echoed signals, considering the interaction between the vessel wall and blood flow. It simulates the behaviors of pulsatile blood flow, modulated by wall motion, passing through a stenosed common carotid artery (CCA). By combining numerical Runge-Kutta method and ultrasound simulation, it offers considerable flexibility in the ability to change mechanical and ultrasonic parameters, such as the stenosis shape and the characteristics of the ultrasonic probe. Furthermore, compared with methods based on CFD software, this model requires fewer computer resources and shorter computing time. Through this model, the successive B-mode, M-mode, and Doppler US images can be obtained efficiently. It would be a useful tool not only for investigating the behaviors of flow field modulated by the wall motion and the changes of ultrasonic images caused by the wall motion but also for quantitative or qualitative evaluation of new ultrasound imaging technology and the estimation method of blood velocity.

This paper is organized as follows. In Section 2, we firstly describe the general workflow of our study. And then, we present the geometric and mathematical model of pulsatile blood flowing through a stenosed vessel, considering fluid-structure interactions (FSI). The rest of this section elaborates on the processes of numerical Runge-Kutta simulation, coupling, and ultrasound simulation. In Section 3, this simulation model was applied to several imaging applications. The B-mode, M-mode, and Doppler US images were obtained. Then, to demonstrate the capability of this model, we made two types of comparisons: here one is among the results with different wall movements, for illustrating the model which can reflect the influence of wall movement on the ultrasound images; and the other is between the numerical and ultrasound simulation results, for demonstrating the similarity between the reference values and results of simulation. In Section 4, the discussion and conclusions of this work are presented.

## 2. Material and Methods

**Figure 1:**
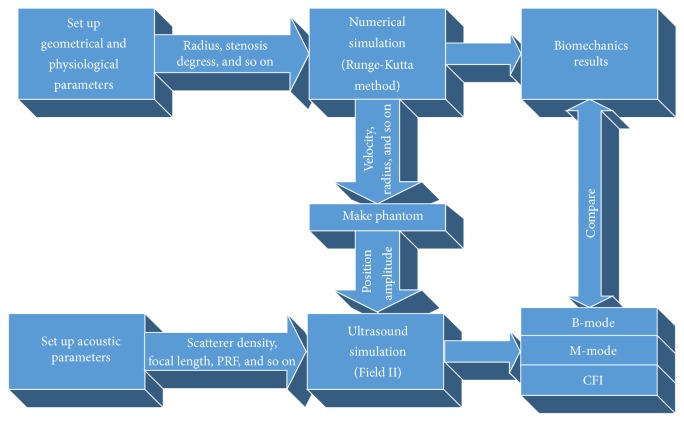
General workflow of the study.

Figure 1 demonstrates the general workflow of our study. Firstly, we defined the geometric and physiological parameters of stenotic arteries in MATLAB, such as the stenosis length, the radius of the artery, the fundamental frequency of blood flow, and Young's modulus. Then, based on these parameters, the fluid-structure interaction model, described by several second-order ordinary differential equations (ODEs), was established. By solving the ODEs with Runge-Kutta method, we obtained numerical solutions of the biomechanical properties of blood flow passing through a constricted vessel, which will be used as input for succeeding ultrasound simulation. Then, the positions and amplitudes of the scatterers were updated, according to the displacements and locations calculated based on obtained blood velocity field and vessel radius changes. Through this combined approach, the acoustic analysis was performed by Field II (an ultrasonic field simulation software). Finally, the B-mode, M-mode, and CFI images were generated and compared with numerical simulation results. Compared with CFD coupled method mentioned, this method requires fewer computing resources and less computing time and is much more convenient for changing model parameters.

### 2.1. Geometrical Model

**Figure 2:**
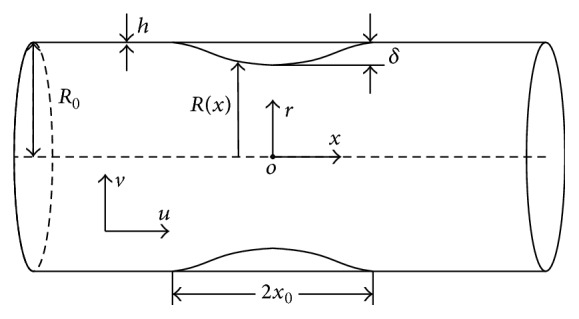
Stenosis geometrical model of carotid artery.

In most cases, the forms of stenosis can be approximately simplified as axisymmetric shapes, although the development of arterial structure does not follow any clearly defined geometrical pattern, and the geometry of the growth is changeable. As shown in Figure 2, the shape of the plaque, in our study, is assumed as the most frequently used cosine curves along the longitudinal direction, which is initially proposed by Young and Tsai [24]. The geometrical parameters of the CCA are created with a radius of 4 mm and an arterial wall thickness of 0.3 mm, which are close to physical values according to recent researches [25, 26]. The length of stenosis is set to be twice of the artery radius, which is corresponding to the average length of the carotid plaques [14]:

(1) Rx=R01−δ2R01+αsin⁡ωt1+cos⁡πxx0,x∈−x0,x0,R0,otherwise.

The geometry of the stenosis, whose mathematical description is given by (1) with the cylindrical polar coordinate system, is assumed to be axisymmetric. The maximum height of stenosis *δ* represents the severity of arterial obstruction. In our study, the motion of the arterial wall, assumed as a sinusoidal vibration, is considered. Accordingly, product term (1 + *α*sin⁡*ωt*) in this formula gives the mathematical expression of the motion of the stenotic arterial wall conducting sinusoidal oscillation at a certain frequency *ω*, while the amplitude of the oscillating arterial stenotic part is determined by a coefficient *α*. To demonstrate the influence of wall movement, four different vibration frequencies (0*∗ω*_0_, 1*∗ω*_0_, 1.5*∗ω*_0_, 2*∗ω*_0_) and three different stenosis degrees (0%, 15%, and 20%) have been investigated, where *ω*_0_ is the fundamental frequency of blood flow. Hence, 0*∗ω*_0_ indicates that the wall oscillating frequency equates to the product of the fundamental frequency of blood flow multiplied by zero, and so on.

### 2.2. Mathematical Modeling

#### 2.2.1. Fluid and Solid Models

In order to simplify the calculation, several relatively unimportant features such as non-Newtonian viscosity, slurry particles in the fluid, and temperature of biological flows may be neglected [27]. Thus, in this study, blood is assumed to be laminar, Newtonian, fully developed, viscous, and incompressible pulsatile flow [28]. For an arterial system, the wave speed of pulsatile blood flow is greater than flow speed, and the radius of the vascular tube is much smaller than the wavelength of the pulse [29]. Consequently, Navier-Stokes Equations can be linearized as follows [30]:

(2) ∂u∂x+1r∂rv∂r=0,∂u∂t=−1ρ∂p∂x+ηρ∂2u∂r2+1r∂u∂r,∂p∂r=∂p∂θ=0.

#### 2.2.2. Wall Modeling

The arterial wall is assumed as isotropic and elastic material obeying Hooke's law. Accordingly, the behavior of periodically pulsatile blood flow and the motion of the arterial wall are interactional and interdependent:

(3) ρw∂2ζ∂t2=E1−σ2∂2ζ∂x2+σR∂ξ∂x−ηh∂u∂r+∂v∂xr=R,ρw∂2ξ∂t2=phE1−σ2ξR2+σR∂ζ∂x−2ηh∂v∂rr=R.

No-slip and no-penetration boundary conditions are supposed between the blood flow and the arterial wall. Therefore, the displacements of the fluid and solid part must be consistent. These coupling conditions are given in

(4) $$\begin{array}{c} {\left.\;u\;\right|}_{r=0}\;\;is\;\;finite,\\ {\left.\;v\;\right|}_{r=0}=0,\\ {\left.\;u\;\right|}_{r=R}=0,\\ {\left.\;v\;\right|}_{r=R}=\frac{\partial \zeta }{\partial t}. \end{array}$$

If the high-order influence of the inertia items of wall and the viscous term of blood are ignored, the equation of wall motion at certain frequency can be approximated as follows [31]:

(5) $$\begin{array}{c} \xi =\frac{pR^{2}\left(1-\sigma^{2}\right)}{Eh}. \end{array}$$

### 2.3. Solutions for the Equations

Because pulsatile blood flow is physically periodic, the parameters of blood flow, such as pressure, pressure gradient, and velocity, can be expressed by a sum of Fourier components in the following:

(6) u=u0+∑i=1Nuiejω0it,

(7) v=v0+∑i=1Nviejω0it,

(8) p=p0+∑i=1Npiejω0it,

where *u*_0_, *v*_0_, and *p*_0_ are stationary items and *u*_*i*_, *v*_*i*_, and *p*_*i*_ are *i*th harmonic items of pulsatile blood flow. *N* is the total number of Fourier decomposition items, which is set to fourteen here for a better approximation. The boundary conditions equations (4) are represented in the form of the following Fourier series:

(9) $$\begin{array}{c} {\left.\;u_{0}\;\right|}_{r=0}\;\;is\;\;finite,\\ {\left.\;u_{i}\;\right|}_{r=0}\;\;is\;\;finite,\\ {\left.\;v_{0}\;\right|}_{r=0}={\left.\;v_{n}\;\right|}_{r=0}=0,\\ {\left.\;u_{0}\;\right|}_{r=R}={\left.\;u_{i}\;\right|}_{r=R}=0,\\ {\left.\;v_{i}\;\right|}_{r=R}=jw_{0}i\zeta_{i}. \end{array}$$

After substituting (8)-(9) into (2) with boundary conditions equations (9), the following set of equations are obtained [32]:

(10) u0=14μ−dp0dxR2−r2,

(11) v0=18μd2p0dx2R2−r22+14μdp0dxRdRdxr,

(12) R4d2p0dx2+dp0dxdRdx=0,

(13) un=1jnω0ρdpndxJ0βnj3/2rJ0βnj3/2R−1,

(14) vn=1jnω0ρr2−1βnj3/2J1βnj3/2rJ0βnj3/2Rd2pndx2−1jnω0ρdRdxJ1βnj3/2RJ20βnj3/2RJ1βnj3/2rdpndx,

where *μ* is the kinematic viscosity of blood, $\beta_{i}=\sqrt{\rho \omega_{0}i/\eta }$, *i* = 1,2, 3, *L*, and *J*_0_ and *J*_1_ are the first kind Bessel functions with zero-order and first-order, respectively. Substituting (5) into (14), the second-order variable coefficient differential equation can be obtained:

(15) 1n2ω02ρR2−1βnj3/2J1βnj3/2RJ0βnj3/2Rd2pndx2+1n2ω02ρdRdxJ12βnj3/2RJ20βnj3/2Rdpndx+R2Bhpn=0.

### 2.4. Numerical Method

In our study, the function ode45 in MATLAB is utilized to solve (15), which is the built-in solver for ODEs and implements a Runge-Kutta method with a variable time step for efficient computation. Firstly, (15) is reduced to a series of first-order equations. After the defined integral span, precision and initial conditions are substituted into these first-order ODEs, the numerical results of pressure harmonic components *p*_*i*_ are obtained. Finally, by substituting *p*_*i*_ into (14) and (15), the parameters, such as pressure, pressure gradient, and velocity of blood flow, can be calculated.

**Figure 3:**
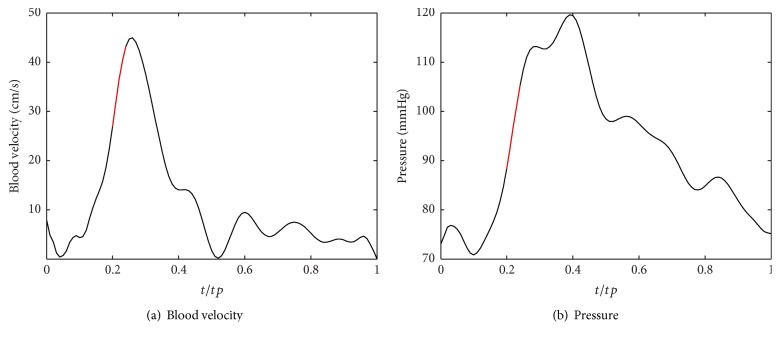
Inlet parameters at *x* = −2*x*_0_ during a cardiac cycle.

Figure 3(a) shows the centerline velocity waveform during one cardiac cycle in the stenotic arterial segment upstream at the position (*x* = 5*R*_0_), which is measured as the ensemble averaged mean velocity waveform from 50 realizations of a normal CCA (the carotid artery without stenosis) by using a portable Doppler ultrasound system (KJ2V2U, Nanjing KeJin Industrial Limited, Nanjing, China) and a 4-MHz transducer [33]. The arterial pressure wave during one cardiac cycle, shown in Figure 3(b), is deduced from the distance between the heart and the CCA. Here, a dimensionless parameter *t*/*tp* is introduced to represent the horizontal direction, where *t* is the specific time point and *tp* is the period of the cardiac cycle. As shown in both figures, the red line segments represent the systolic acceleration phase (*t*/*tp* = 0.22–0.26). The parameters used in our computation are listed in Table 1.

### 2.5. Simulating Ultrasound Coupling Numerical Simulation

In this study, the ultrasound simulation is conducted with the Field II software which is created by Jensen [34, 35]. Based on linear systems theory, this software simulates ultrasound field using the spatial impulse response method proposed by Tupholme and Stepanishen [36, 37]. Since any transducer can be simulated by splitting the aperture into some kind of small shaped subapertures, and any transducer excitation and apodization can be included in the calculation, it is feasible to obtain realistic simulated ultrasound images [38].

In Field II program, the tissue is treated as a collection of randomly distributed point scatterers. Each ultrasound echoed beam, collected by the receiving transducer, can be calculated by summing the responses from these scatterers, and the scattering strength is determined by the density and speed of sound perturbations in the tissue [39]. As shown in Figure 1, the ultrasound simulation is performed based on the numerical outputs such as the velocity vector of blood flow, vessel radius, and other geometrical and physiological parameters. Through this combined approach, these scatterers in an ultrasound simulation move with velocities generated by the aforementioned numerical simulation. In this study, a full coupling method is used to capture the dynamic arterial flow and the estimated velocity field. Thus, the position of the scatterers can be updated at any moment over the cardiac cycle, and the dynamic ultrasound B-mode, M-mode, and CFI images can be achieved.

#### 2.5.1. Setup Phantom

In this study, the phantoms are angled 45° with respect to the ultrasound beam which is in the axial direction. Considering the length of ultrasound simulation time, the scatterer density is set to ten scatterers per resolution cell, although it is related to the resolution of the images. According to its specific location (blood, arterial wall, and surrounding tissues) and simulation type (B-mode, M-mode, and CFI), these point scatterers are given different amplitude values whose distribution follows a Gaussian distribution [40]. The amplitude values used in the simulations are listed in Table 2.

To combine the numerical and ultrasound simulations, several works must be done. First of all, the temporal and spatial interpolations of outputs derived from the Runge-Kutta simulation are indispensable because of the difference of resolution. Secondly, the coordinate of every randomly distributed scatterer must be mapped onto the spatially interpolated coordinate of the obtained velocity vector. This operation will inevitably introduce approximate error. In this way, the radial and axial velocities of each scatterer in ultrasound simulation are obtained. Then, the new position of any scatterer can be acquired as

(16) rn=rn−1+v×Δt,xn=xn−1+u×Δt,

where *r*_*n*_, *x*_*n*_ and *r*_*n*−1_, *x*_*n*−1_ are the radial and axial coordinates of the scatterer at current and previous time step, respectively, *u*, *v* are the axial and radial velocity of the mapped scatterer, and Δ*t* is the emitting interval of successive ultrasound beams.

For tissue motion simulation, these scatterers imitate wall and tissue movement only with radial velocity. The displacement within Δ*t* can be obtained from the interpolated output of the Runge-Kutta simulation. Consequently, the updated radius of scatterer in the wall or tissue is described as

(17) $$\begin{array}{c} {radius}_{n}={radius}_{n-1}+\Delta radius\times \left(\;1-\frac{{radius}_{n-1}}{R\_range}\;\right), \end{array}$$

where *R*_range is the radial scan area of the simulation.

Owing to the time-varying behavior of blood flow, files including the position and the amplitude of each scatterer point will be generated after each update. Thereby, a large number of files are involved here, and the exact number depends on the pulse repetition frequency.

#### 2.5.2. Ultrasound Simulation

With the help of Field II software, the generated phantom files are then used to calculate RF lines individually for each different imaging direction and for each time step. Further, by representing these calculated amplitudes of echo beams with brightness and assembling these sequential signals, B-mode and M-mode images are obtained. To simulate velocity of blood flow or tissue motion, we applied an autocorrelation method, which was introduced by Kasai et al. [41] for ultrasound application. This approach computes mean axial velocity based on the phase shift of the center frequency of the RF signal. It is mathematically described as

(18) vz=c4πf0TPRF·tan−1∑i=1N−1yixi−1−xiyi−1∑i=1N−1xixi−1−yiyi−1,

where *c* is the wave velocity, *f*_0_ is the central frequency, *T*_PRF_ is the pulse repetition period, *x* and *y* are the real and imaginary parts of echo signal, respectively, and *N* is the number of samples, which is related to accuracy. Thus, an estimated velocity in one direction is obtained. Doing this for all directions in an image yields a mapping of the velocity, displayed in color flow mapping (CFM) systems. The acoustic parameters of ultrasound simulation in Field II software are summarized in Table 3.

## 3. Results and Discussions

This section presents several applications of this model, which includes two parts. The first part illustrates the applications related to the movement of the vessel wall. In this part, B-mode and M-mode figures were generated and compared. Furthermore, we obtained the wall displacement with an image segmentation technique and compared it with reference values. The second part deals with applications related to the blood velocity estimation. In this part, CFI figures and velocity profiles for all cases were compared with each other and to reference numerical results. The simulations for all cases were performed in MATLAB 8.3 and on a workstation with two Intel Xeon 4 cores 64-bit processors (1.8 GHz) and 16 G RAM.

### 3.1. Wall Movement Application

#### 3.1.1. B-Mode Simulation

**Figure 4:**
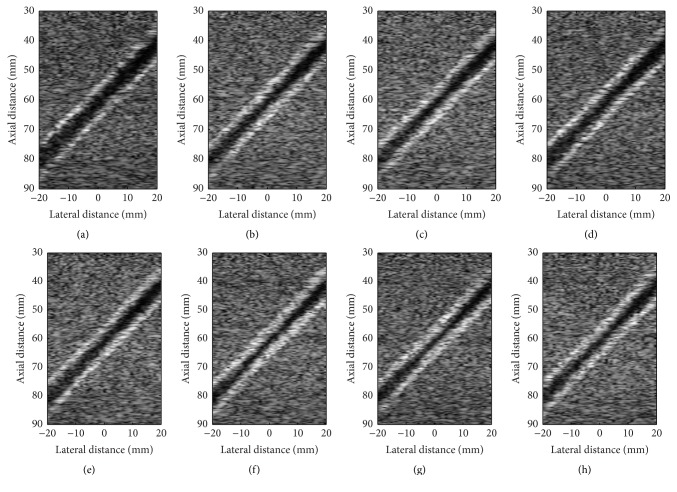
Simulated B-mode images with 15% (a–d), 25% (e–h) stenosis degree, and 0*∗ω*_0_ (a, e), 1*∗ω*_0_ (b, f), 1.5*∗ω*_0_ (c, g), and 2*∗ω*_0_ (d, h) wall oscillating frequencies when *t*/*tp* = 0.26, respectively.

Figure 4 shows the B-mode images which are samples extracted from the simulated frames with different physical factor when the inlet velocity almost reaches the maximum (*t*/*tp* = 0.26). Each row illustrates arterial stenosis with different wall oscillating frequencies and same narrow degree, while each column illustrates that with different stenosis degrees and the same frequency.

It can be clearly observed in these subfigures that the arterial wall and stenosis parts are hyperechoic, demonstrated with a brighter color, while blood flow is hypoechogenic, illustrated with black. Thus, the narrow degree of the blood vessel can be detected from the hyperechoic area of these subfigures. Further, the differences can be easily found by comparing these subfigures with each other. As shown, we can learn that the position of vessel wall changes with the wall oscillating frequencies. However, the changes are imperceptible when the contracted degree is not serious, in spite of the different wall oscillating frequencies. Nevertheless, it gets more noticeable with the increase of narrow severity. For example, as shown in Figure 4(f) (with 25% stenosis, 1*∗ω*_0_ wall oscillating frequency), the hyperechoic area of the vessel is larger than that with other frequencies. Hence, these B-mode images can qualitatively demonstrate the movement of the vessel wall. Moreover, this model can generate successive B-mode images, which will dynamically show the wall movement.

#### 3.1.2. M-Mode Simulation

**Figure 5:**
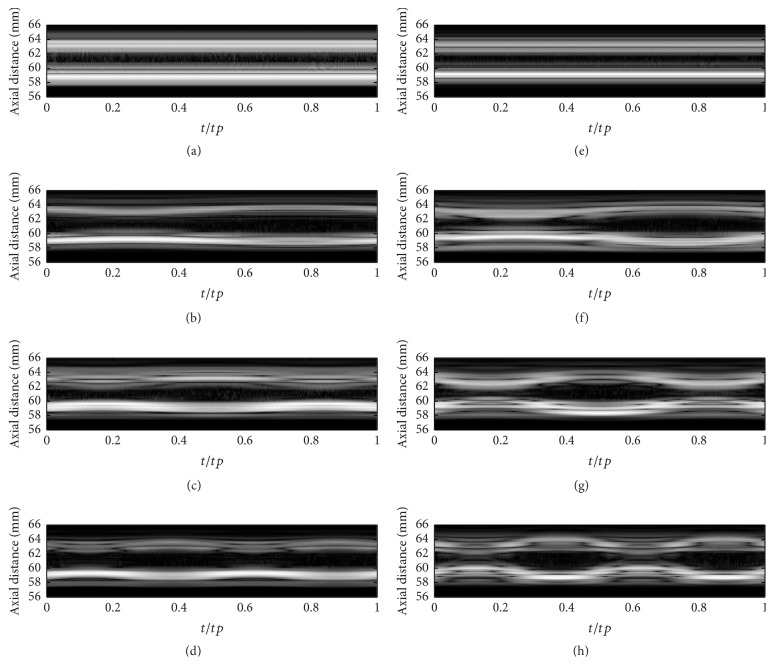
Simulated M-mode images with 15% (a–d), 25% (e–h) stenosis degree, and 0*∗ω*_0_ (a, e), 1*∗ω*_0_ (b, f), 1.5*∗ω*_0_ (c, g), and 2*∗ω*_0_ (d, h) wall oscillating frequencies during a cardiac cycle.

As Figure 5 shows, the simulated M-mode images for different cases illustrate the movement of the vessel wall during one whole cardiac cycle. We just chose the echoed RF data at the axial central position of stenosis (*x* = 0) to show the wall movement over time. These M-mode images clearly demonstrate that the wall vibrates almost in a sinusoidal pattern with a certain frequency, which coincides with the mathematical formula defined by (1). Moreover, it can be learned from these figures that the vibration amplitudes increase with the narrow degree. However, the vessel dilatation and constriction caused by pulsatile blood flow are hardly discerned in these figures.

**Figure 6:**
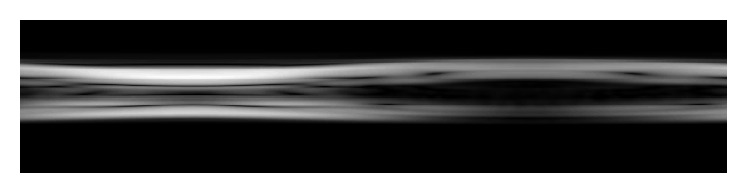
SRAD processed M-mode image based on Figure 5(f) (stenosis degree = 25%, *ω* = 1*∗ω*_0_).

A segmentation algorithm, speckle reducing anisotropic diffusion (SRAD) [42], was used to demonstrate the application of this model. Here, we only chose Figure 5(f) to show the result of SRAD process. As shown in Figure 6, the speckle noise is reduced, and wall boundaries are sharper. Then, the wall displacement can be easily acquired from these processed M-mode images, based on a simple thresholding method.

**Figure 7:**
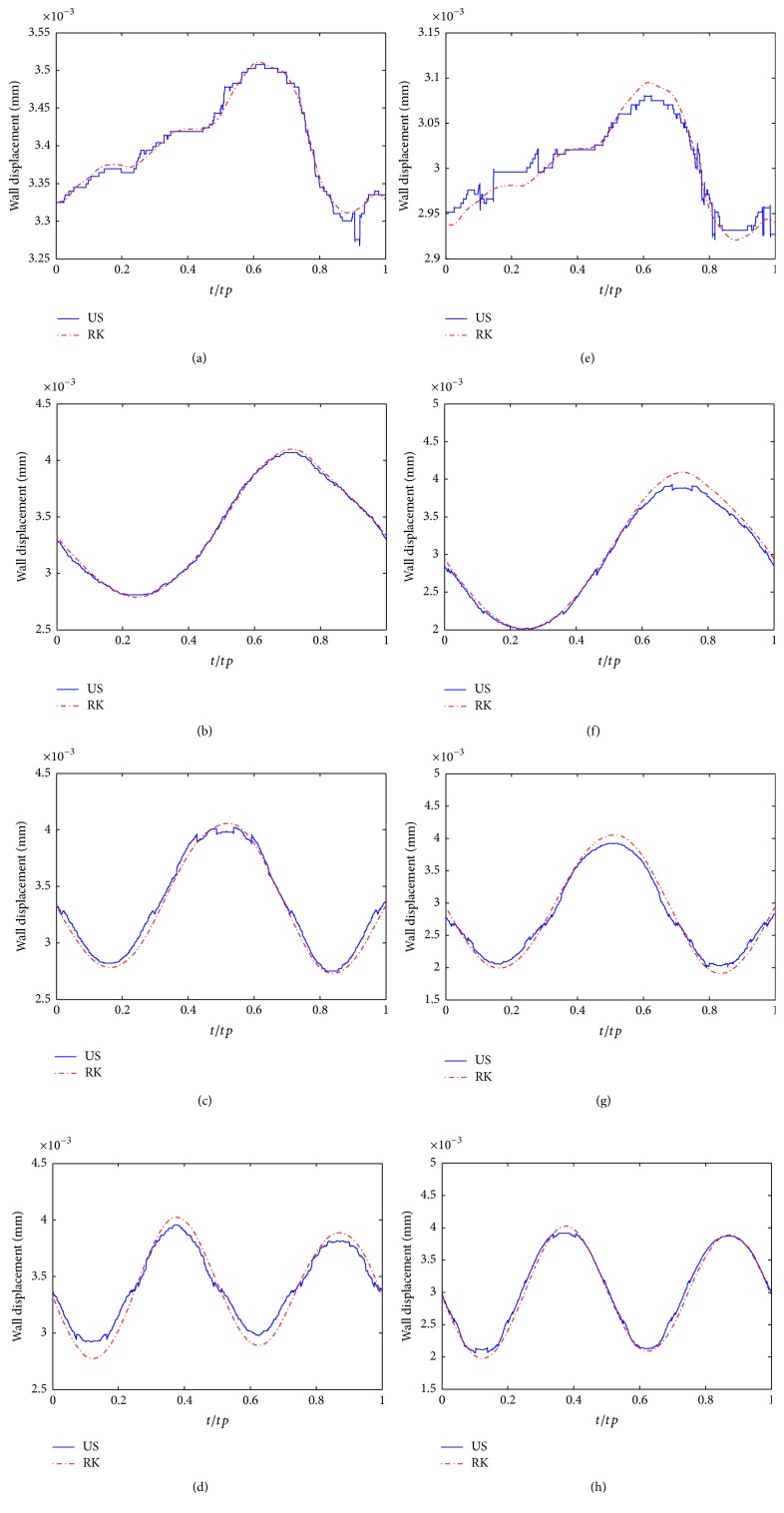
Comparisons of the wall displacement between the data from Runge-Kutta numerical simulation and ultrasound simulation with 15% (a–d), 25% (e–h) stenosis degree, and 0*∗ω*_0_ (a, e), 1*∗ω*_0_ (b, f), 1.5*∗ω*_0_ (c, g), and 2*∗ω*_0_ (d, h) wall oscillating frequencies during a cardiac cycle.

Figure 7 demonstrates the wall displacement from the Runge-Kutta numerical and the ultrasound simulation. The blue curves in these subfigures illustrate that the wall displacements got from the ultrasound simulation, which are the average values of 30 simulation results. All these curves were obtained from SRAD processed output with the same threshold. As shown, the ultrasound simulation results are in agreement with red curves, which depict the data got from the Runge-Kutta method. However, there is a small underestimation in some subfigures, such as in Figures 7(c) and 7(d). For further quantitative analysis, we calculate the mean and the maximum relative errors between the two types of wave forms and list them in Table 4. As shown, the mean error in all cases is minor, while the maximum relative error is 7.48% (stenosis degree = 25%, *ω* = 2*∗ω*_0_).

### 3.2. Flow Velocity Simulation

**Figure 8:**
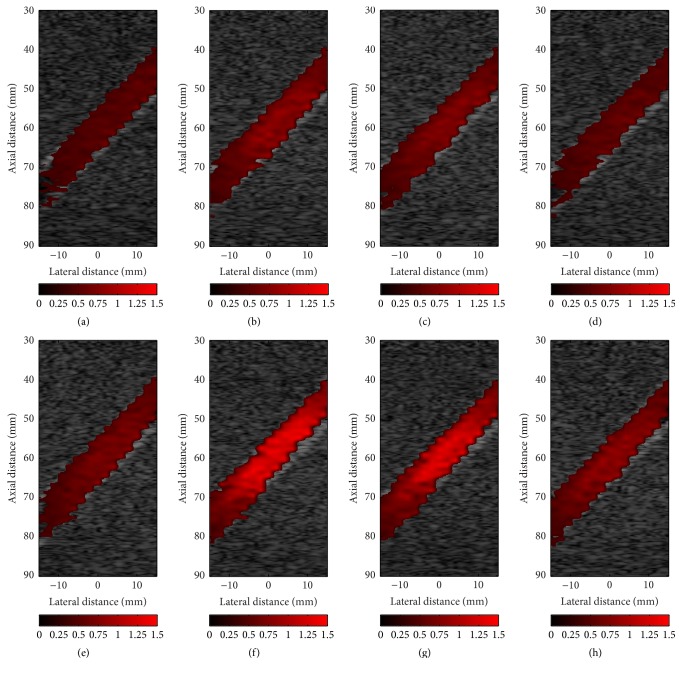
Simulated CFI images with 15% (a–d), 25% (e–h) stenosis degree, and 0*∗ω*_0_ (a, e), 1*∗ω*_0_ (b, f), 1.5*∗ω*_0_ (c, g), and 2*∗ω*_0_ (d, h) wall oscillating frequencies during systolic acceleration phase (*t*/*tp* = 0.22–0.26).

As mentioned before, we can estimate the blood flow velocity from autocorrelation. The parameters of ultrasound simulation in Field II software are listed in Table 3.  Figure 8 demonstrates color flow images (CFI) of the systolic acceleration phase (the red line segment shown in Figure 3, *t*/*tp* = 0.22–0.26) of blood flowing through the vessel with different stenosis degree and wall oscillating frequencies. Several features can be obtained by comparing these figures with each other. First, the maximal velocity of blood flow in the vessel with oscillating wall is greater than that in tubes without wall oscillating (the first column, *w* = 0*∗w*_0_). Second, the maximum estimated velocity of blood decreases along with the increase of wall oscillating frequency. In particular, when the wall oscillating frequencies equate to the fundamental frequency of blood flow, these estimated maximum velocities (as shown in the second column of Figure 8) are higher than the maximum velocity with the same stenosis. Moreover, by comparing the rows, we can see that the increased value of the estimated velocity with a higher degree of stenosis is much more striking than that with lower degrees.

**Figure 9:**
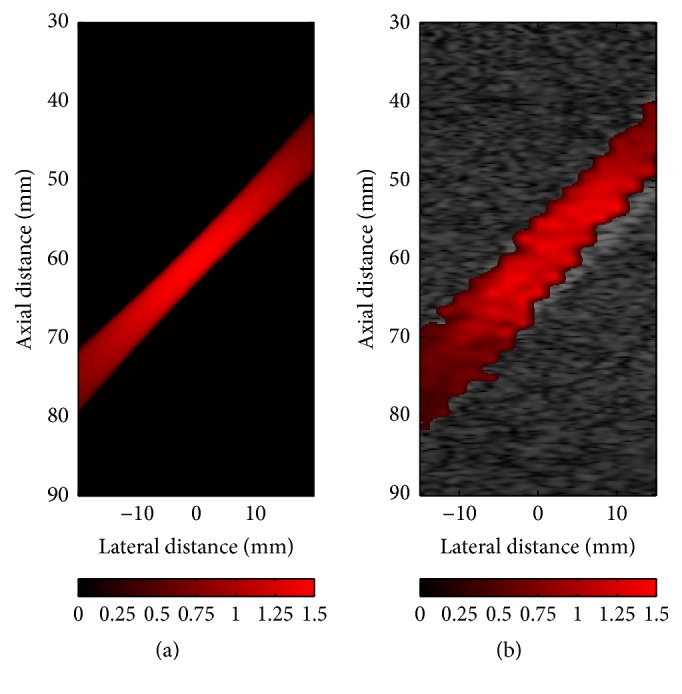
CFI images with data from Runge-Kutta method (a) and from ultrasound simulation (b) when *t*/*tp* = 0.22–0.26.

As Figure 9 shows, the color flow image with data from the Runge-Kutta numerical simulation (a) is generated and compared with that obtained from ultrasound simulation (b). In spite of the fact that the simulated ultrasonic CFM image is more irregular to the velocity estimator statistics, it can be observed from these color flow images that the velocity field acquired from ultrasound simulation agrees well with the Runge-Kutta simulation results. For further quantitative analysis, the value of each point in the ultrasound simulated velocity field is compared with that of the corresponding reference velocity.

**Figure 10:**
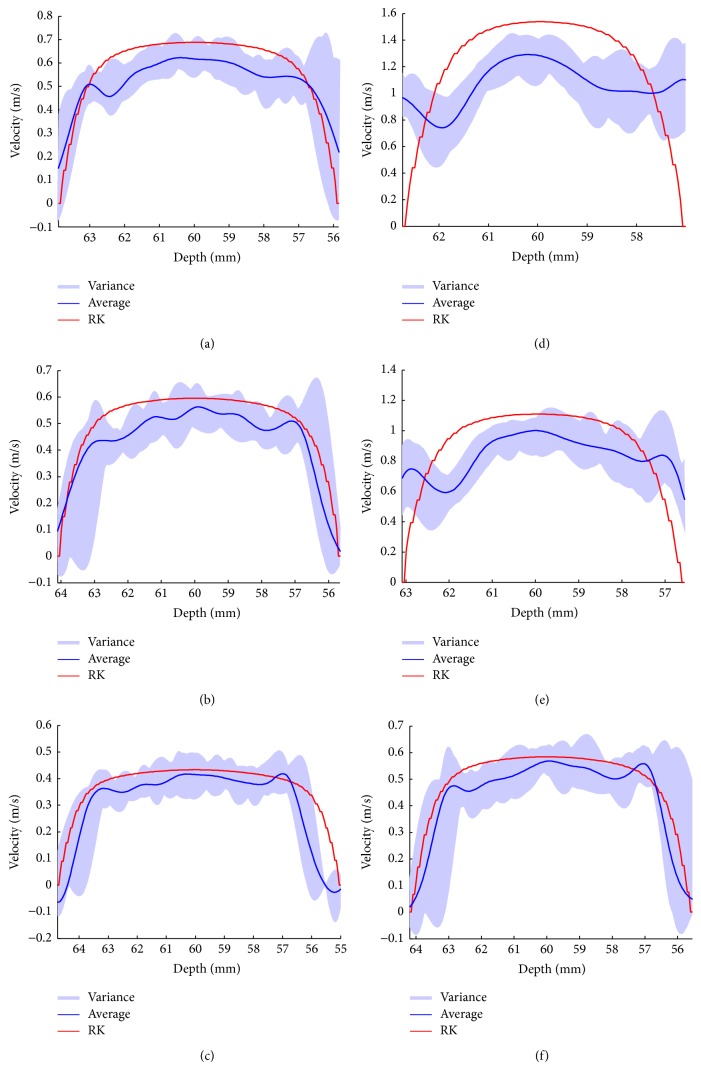
Velocity profile of blood flow with 15% (a–c), 25% (d–f) stenosis degree, and 1*∗ω*_0_ (a, d), 1.5*∗ω*_0_ (b, e), and 2*∗ω*_0_ (c, f) wall oscillating frequencies.

The velocity profiles obtained from the lateral center are illustrated in Figure 10, in which the blue curves depict the average velocity of 30 autocorrelation results while the red curves represent the reference velocity, and the light blue shaded areas in these figures represent the variance of obtained velocity. As can be observed, the ultrasound simulated velocity profile shows the almost same shape as the reference velocity. However, the difference between the two curves increases with the growth of the maximum velocity because of Nyquist's limit. In general, the simulated velocity is underestimated, especially near the central area. Furthermore, because these ultrasonic CFM figures (Figure 8) are illustrated without filtering out low-frequency and high-amplitude clutters echoed by slowly moving tissues, the estimated blood flow velocities, especially higher velocities, are contaminated. For example, as Figures 10(d) and 10(e) show, the estimated velocities in the area near the wall significantly differ from the reference velocities. The presence of these discrepancies reflects the features of real signals in clinic, which is the reason why the clutter filter is indispensable. Meanwhile, it suggests that our model can be used to assess the clutter filter.

**Figure 11:**
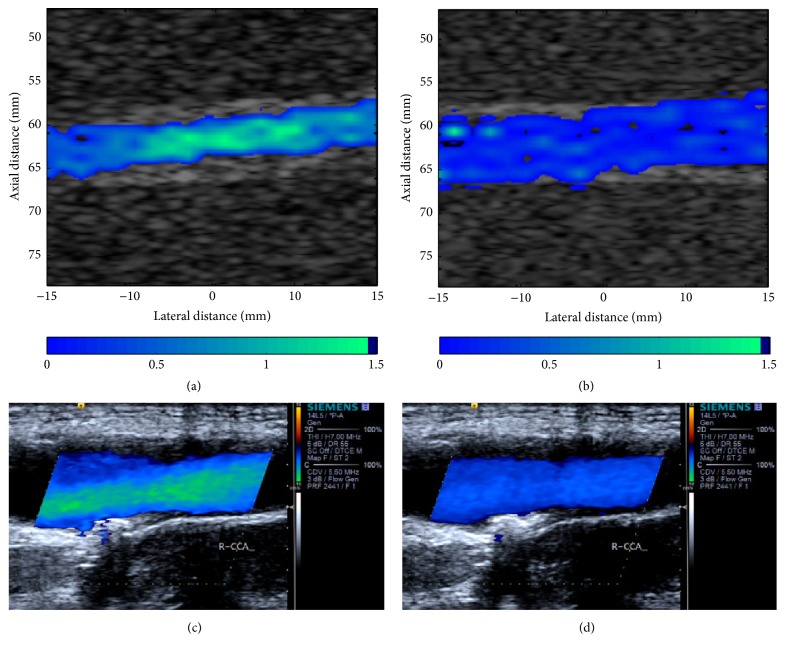
CFI images from ultrasound simulation (a-b) with 25% stenosis degree and 1*∗ω*_0_ oscillating frequency and from clinical data (c-d) obtained from a right CCA with a moderate stenosis, in systole (a, c) and in diastole (b, d).

To further demonstrate capability of this model, we compared the simulated CFM figures with clinical data, as shown in Figure 11. The clinical CFM figures were obtained from the right CCA of a patient with a mild plaque. The angle between the probe and artery is about 120°, so the blood in Figure 11 is displayed in blue. Figures 11(a) and 11(c) illustrate CFM images in systole, and the middle parts in the vessels of both images are light blue which means the blood with higher velocity (the maximum blood velocity in Figure 11(c) is about 1.4 m/s.), while the blood velocity of outer parts is lower, represented in deeper blue. Figures 11(b) and 11(d) represent images in diastole. The blood in both figures is deep blue and there is a little change in color, which means the blood is stable and the velocity is lower. By comparison, the results illustrate that the simulated images show good consistency with clinical ones.

### 3.3. Discussions

With this model, as shown in Figures 4, 5, and 8, we can easily obtain B-mode, M-mode, and CFM ultrasound images. From these generated images, by comparing with different cases, it is clear that this model can qualitatively reflect the impact of wall motion on the blood field. Moreover, it can generate B-mode figures at any time, or successive B-mode and CFM figures during any period, as well as M-mode figures at any location. Accordingly, these figures can dynamically show the motions of blood flow and vessel wall, which is of more help in understanding the behaviors of blood flow modulated by wall motion. Meanwhile, the results shown in Figure 7, Table 4, and Figures 9 and 10 demonstrate that the ultrasound simulated results provide a good match with corresponding theoretical values. Furthermore, by comparison with clinical data, Figure 11 demonstrates that this model can simulate realistic ultrasonic figures. Therefore, we suggest that this model can be used as an analysis tool for quantitative evaluation of new ultrasound imaging technology and the estimation method of blood velocity, such as image segmentation or clutter filter.

In view of the fact that the object of this study is the stenosed CCA, the shape of the vessel in this study is 3D tubular instead of bifurcate or curved. Further, as mentioned, the main aim of this model is to generate ultrasound echoed signals, which can be used for investigating the changes of ultrasound images caused by wall motion and for evaluating ultrasound imaging and the estimation method of blood velocity. Thus, for this model, the most important requirement is the ability to accurately reflect the behaviors of blood flow modulated by wall motion. This is demonstrated by those abovementioned figures and comparison results. Moreover, with the tubular geometry, this model will not need to deal with complicated flows and wall motions caused by complex shapes, which will increase the complexity of ultrasound imaging and estimation method of blood velocity. Furthermore, the pipe-like shape will simplify the simulation model and decrease the calculation time.

Based on the velocity field obtained from the Runge-Kutta numerical simulation, Field II software was used to generate B-mode, M-mode, and CFI images to visualize the motions of the blood flow and the vessel wall. As mentioned, for this fully coupled simulation method, the amplitude and position of every scatterer need to be updated after each RF calculating, which accordingly make the ultrasound simulation results show good agreement with the reference model. However, a great amount of computation time is undoubtedly involved, which is related to the scatterer density, the pulse repetition frequency, and scan line number. For example, the simulation time for any CFM case was almost four hours when fprf = 16 k and scan line = 20, while it was reduced to about half an hour when parallel computing technology was used on a multicore workstation.

To a certain extent, the implementation in our study is similar to the ones proposed by Balocco et al. and Swillens et al., in which the biomechanics and ultrasonic analysis are integrated based on US simulation software (Field II). The computational fluid dynamics (CFD) method was used in their studies to generate a new amplitude and position for each scatterer, while our model uses the Runge-Kutta method. Compared with CFD-US coupled method, this model needs fewer computer resources and less computing time and is much more convenient for changing model parameters. Furthermore, by taking the movement of tissue into account, this model can be used not only as an evaluation tool, but also as a simulation environment for investigating the interaction between the blood flow and arterial wall. For example, in Section 3, the comparison between results of all cases illustrates that the movement of the stenosed vessel wall will significantly affect the behavior of blood flow. It can be listed as follows. (1) The vibrating motion of the arterial wall increases contraction and further increases the maximal velocity of blood flow. (2) The closer to blood fundamental frequency the wall vibrating frequency is, the higher peak velocity becomes. (3) The increased value of blood velocity with a higher stenosis degree is much striking than that with lower degrees.

In this study, the arterial wall motion is treated as a simple sinusoidal motion, while a more realistic motion should be taken into consideration in future research. For example, the wall motion can be treated as a complex movement influenced by breathing and the heart beating. Moreover, due to the assumption of taking arterial wall as isotropic and elastic material obeying Hooke's law, the model cannot accurately depict the behaviors of all biological tissues. Hence, considering the anisotropy of vessel membrane and the viscoelasticity of the vessel wall may improve this model. However, although some simplifications have to be done in this study, this model is explicit on quantitatively assessing the ultrasound image changes induced by the changes of the vessel wall movement, such as the wall vibrating frequency, amplitude, and stenosis degree.

## 4. Conclusion

An ultrasound simulation model for the pulsatile blood flow, modulated by the motion of a stenosed vessel wall, is presented in this paper. Considering fluid-structure interaction, blood pulsatility, stenosis of the vessel, and arterial wall movement caused by surrounding tissue's motion, it can generate more realistic ultrasonic signals. This proposed model couples ultrasound and numerical Runge-Kutta method and is characterized by its considerable flexibility in the ability to change mechanical and acoustic parameters. The simulated results show greater consistence with corresponding theoretical values and reflect the influence of wall movement on the flow field. It can serve as an effective tool not only for investigating the behavior of blood flow field modulated by wall motion but also for quantitative or qualitative evaluation of new ultrasound imaging technology and estimation method of blood velocity.

## Figures and Tables

**Table 1:** Parameters used in computation.

*R*_0_ (mm)	*ρ* (kg/m^3^)	*E* (Pa/m^2^)	*tp* (s)	*w*_0_	*σ*	*x*_0_ (mm)	*h* (mm)	*μ* (Pa·s)
4	1050	978*∗*10^3^	2/3	3*π*	0.5	40	0.3	3.5*∗*10^−3^

**Table 2:** Amplitude value of backscattering in different area.

Area	Wall	Blood	Tissue
B-mode	50	5	10
M-mode	50	5	0
CFI	0	5	0

**Table 3:** Setup parameters of ultrasound simulation.

Setup parameters	Simulation type		
	B-mode	CFI	M-mode
Center frequency (MHz)	3	5	3.75
Number of elements	196	196	196
Active elements	64	64	64
Kerf (mm)	0.05	0.05	0.03
Height (mm)	5	5	5
Focus (cm)	7	4	4
Dynamic receive focusing	Yes	Yes	Yes
Aperture	Linear	Linear	Convex
Excitation	Sin	Sin	Sin
Pulse periods	2.5	2.5	2.5
PRF max (KHz)	8	1	3.5

**Table 4:** The mean and maximum relative errors of wall displacement.

Relative errors		Wall vibrating frequency			
Stenosis degree	Error	0*∗ω*_0_	1*∗ω*_0_	1.5*∗ω*_0_	2*∗ω*_0_
15%	Average	0.17%	0.50%	1.55%	2.45%
	Max	1.52%	1.80%	4.19%	5.61%

25%	Average	0.33%	1.88%	3.28%	2.19%
	Max	0.98%	5.37%	7.24%	7.48%

## Nomenclature

*R*(*x*): The radius of stenosed artery
*R*_0_: The healthy artery radius
*h*: The thickness of the healthy arterial wall
*r*: The radial coordinate
*x*: The axial coordinate
*θ*: The angular coordinate
*δ*: The maximum height of stenosis
*α*: The oscillating amplitude of wall
*ω*: The oscillation frequency of stenosis
*x*_0_: The half-length of the stenosis
*u*: The axial component of velocity
*v*: The radial component of velocity
*p*: The pressure of blood
*ρ*: The blood density
*η*: The blood viscosity
*t*: The time
*ξ*: The radial vascular displacements
*ζ*: The axial vascular displacements
*ρ*_*w*_: The wall density
*E*: Young's modulus
*σ*: Passion's ratio.
